# Brain near-infrared study of upstairs movement after anterior cruciate ligament reconstruction

**DOI:** 10.3389/fneur.2024.1500579

**Published:** 2025-01-07

**Authors:** Ziyuan Cao, Hao Zhang, Xipeng Wu, Yuxuan Zhang, Jiangli Yu, Wei Li

**Affiliations:** ^1^School of Special Education and Rehabilitation, Binzhou Medical University, Yantai, China; ^2^Department of Rehabilitation Medicine, Shandong Provincial Third Hospital, Jinan, China; ^3^Department of Neurology, The Second Medical Center and National Clinical Research Center for Geriatric Disease, Chinese PLA General Hospital, Beijing, China; ^4^Department of Rehabilitation, Binzhou Medical University Hospital, Binzhou, China

**Keywords:** ACLR, fNIRS, rehabilitation, sports injury rehabilitation, brain function

## Abstract

**Objective:**

After anterior cruciate ligament reconstruction (ACLR), patients undergo specific changes in body and specific brain functions, which stem from neuroplasticity. In this study, we employed functional near-infrared spectroscopy (fNIRS) to investigate the characteristics of brain activation in patients after ACLR during a repetitive upstairs task, and compared them with healthy individuals. We aimed to provide a new theoretical basis for the changes in brain function after ACLR and neurorehabilitation of sports injuries.

**Methods:**

A total of 27 patients who undergoing right ACLR and 27 healthy controls participated in the study. We utilized fNIRS to collect hemodynamic data from the frontal and parietal cortices of both groups during a repetitive upstairs task. The Lysholm scale assessment was conducted prior to the commencement of the task. Compare the functional characteristics of the brain in post-operative patients and healthy subjects during upstairs tasks, and examine the functional differences between the two groups.

**Results:**

(1) Patients undergoing ACLR demonstrated a significant negative change in *β*-value for Channel 25 (*t* = 4.0461, *p* = 0.0067) during the repetitive upstairs task. (2) In contrast, the healthy control group exhibited a significant increase in *β*-value across Channel 6 (*t* = −3.0489, *p* = 0.0066), Channel 7 (*t* = −4.5723, *p* = 0.0002), Channel 8 (*t* = −3.0089, *p* = 0.0072), Channel 13 (*t* = −2.8789, *p* = 0.0096), Channel 20 (*t* = −3.4200, *p* = 0.0029), and Channel 33 (*t* = −2.6974, *p* = 0.0143) during the task. (3) When compared to the healthy control group, ACLR patients exhibited a significant negative change in *β*-value for Channel 25 (*t* = 2.7583, *p* = 0.0089), and Channel 33 (*t* = 3.0618, *p* = 0.0040).

**Conclusion:**

Patients with ACLR exhibited a significant negative activation in a specific brain region during upward stair movements. In contrast, healthy individuals demonstrated activation in two particular brain areas during the same task. Interventions targeting these brain regions may represent a novel rehabilitation approach. This provides a theoretical basis for incorporating fNIRS into the rehabilitation assessment of patients undergoing ACLR. In conclusion, this study provides a theoretical framework for potential interventions and assessments of brain regions following ACLR.

## 1 Introduction

Anterior cruciate ligament (ACL) injuries are a common condition with a rising incidence in American professional football (1). Likewise, the incidence of ACL injuries in individual’s daily lives is also increasing annually. These injuries can significantly impact athletic ability and long-term health (2).

Previous studies have documented early successful recovery following ACL reconstruction (ACLR). However, recent studies indicate that conservative treatment can also be viable after ACL injury. While most patients resume cutting motions after ACLR, the rates of ipsilateral reinjury and contralateral ACL injuries continue to rise (3). From a medical perspective, subsequent knee damage or the need for further knee surgery represents a relatively unsuccessful treatment approach (4). In addition, although most male professional athletes undergoing ACLR can return to athletic participation within 1 year postoperatively, their long-term participation rates remain unknown (5).

Current research on the mechanisms of non-contact knee injuries primarily focuses on the investigation of biomechanical and neuromuscular characteristics (6). However, recent studies have indicated concomitant changes in brain function among patients who have undergone ACLR.

Neuroplasticity refers to the ability of the central nervous system (CNS) to adapt to external (environmental) and internal (anatomical) factors. These adaptations may include changes in the overall cognitive strategies, recruitment of different neural circuits, or amplification or reduction in the involvement of certain connections or brain regions (7). The utilization of electroencephalography (EEG) for knee position matching and force matching tasks has revealed altered neurocognitive processing related to sensory integration and attentional modulation in patients with ACL reconstruction (8, 9). Functional magnetic resonance imaging (fMRI) studies have demonstrated reduced activation in several sensory-motor regions and increased activation patterns in the visual and cortical motor centers of patients with ACLR (10, 11), along with increased activity within the contralateral motor cortex and supplementary motor areas. Monitoring studies of ACLR athletes who returned to play have indicated consistent changes in their brain activity (8). This suggests that patients undergoing ACLR experience more specific changes in brain function, implying that optimizing brain function post-ACLR can be a rehabilitation goal.

To accurately specify the brain regions that undergo functional changes after ACLR, clinicians need to detect functional brain changes in patients undergoing ACLR during more realistic and natural tasks. Numerous studies have confirmed that the use of fNIRS technology effectively assesses and quantifies the hemodynamic responses across different brain regions during motor tasks (12, 13). FNIRS lie in its non-invasive nature and its ability to measure brain responses during natural movements, making it an ideal tool for investigating neural responses in motor tasks. In contrast, traditional neuroimaging techniques, such as fMRI and EEG are often constrained by technical limitations that hinder effective measurement during movement, while fNIRS overcomes this limitation.

Going upstairs is an unavoidable and challenging daily requirement in modern life. At the same time, walking up stairs is an important rehabilitation exercise after ACLR, as it can increase muscle strength and endurance, improve joint range of motion and flexibility, promote the recovery of knee function, and enhance balance and coordination, cardiorespiratory fitness, as well as increase self-confidence and independence. However, few previous studies have investigated brain function during upstairs tasks in patients with ACLR. Therefore, this study aims to utilize fNIRS to monitor the activation characteristics of brain function in patients with ACL injuries while performing repetitive upstairs tasks. Additionally, the study seeks to observe differences in brain activity between these patients and healthy individuals. The findings are intended to provide new evidence regarding changes in brain function following ACLR and to offer a theoretical basis for neural rehabilitation in this context.

## 2 Methods

### 2.1 Participants

With the approval of the Ethics Committee of the Binzhou Medical University Hospital (under the Ethical Approval Number KYLL-2022-112), we included 27 patients (24 males, 3 females; mean age: 25.6 years) undergoing right ACLR and 27 healthy individuals (23 males, 4 females; mean age: 25.8 years) in this study (detail in Table 1), and all patients signed an informed consent form.

**Table 1:** Participant demographics.

Parameters	ACLR group	Healthy control group	*p*
Age (years)	25.6 ± 2.3	25.8 ± 2.8	0.672
Gender (M/F)	24/3	23/4	0.685
Body mass index (BMI)	25.1 ± 3.5	24.9 ± 2.8	0.462
Limb dominance	26R, 1L	26R, 1L	
Time from surgery	4.6 ± 1.4		

Data in the table are means ± standard deviation and percentages. ACLR, anterior cruciate ligament reconstruction.

All patients met the following inclusion criteria: (1) age 18–45 years; (2) arthroscopically complete ACL rupture; (3) preoperative examination confirming the absence of significant osteoporosis or joint degeneration; and (4) simple ACL injuries with no or only first-degree cartilage damage and no meniscus damage. Exclusion criteria were as follows: (1) arthroscopically confirmed ACL injury; (2) conscious patients; (3) complications of serious cardiac, pulmonary, hepatic, renal dysfunction, or other serious physical diseases; (4) presence of obvious osteoporosis or other diseases; (5) presence of more than 2 degrees of the cartilage damage or meniscus; (6) presence of implanted metal devices in the body, such as a cardiac pacemaker or a cranial metal; and (7) individuals with severe cervical spine pathology, including severe cervical cone stenosis and cervical spine instability.

### 2.2 Clinical scale assessment

The Lysholm scale is a widely utilized questionnaire for assessing the functional status of the knee, particularly in the context of rehabilitation following sports injuries and surgical interventions. This scale comprises eight items that evaluate various aspects, including pain, knee stability, swelling, locking sensations, the ability to ascend and descend stairs, squatting ability, the need for support, and limitations in general activities. Before the patient undertook the upstairs task, a series of assessments utilizing Lysholm score were conducted. Additionally, the patient’s Lysholm score prior to the injury was meticulously documented.

### 2.3 Study design and settings

All participants were asked to stand naturally with both upper limbs relaxed and naturally positioned at the sides of the body upon entering a quiet and light-avoiding stairwell (Stair height 16 cm, width 29.7 cm). Ambient light and noise levels were reduced.

**Figure 1:**
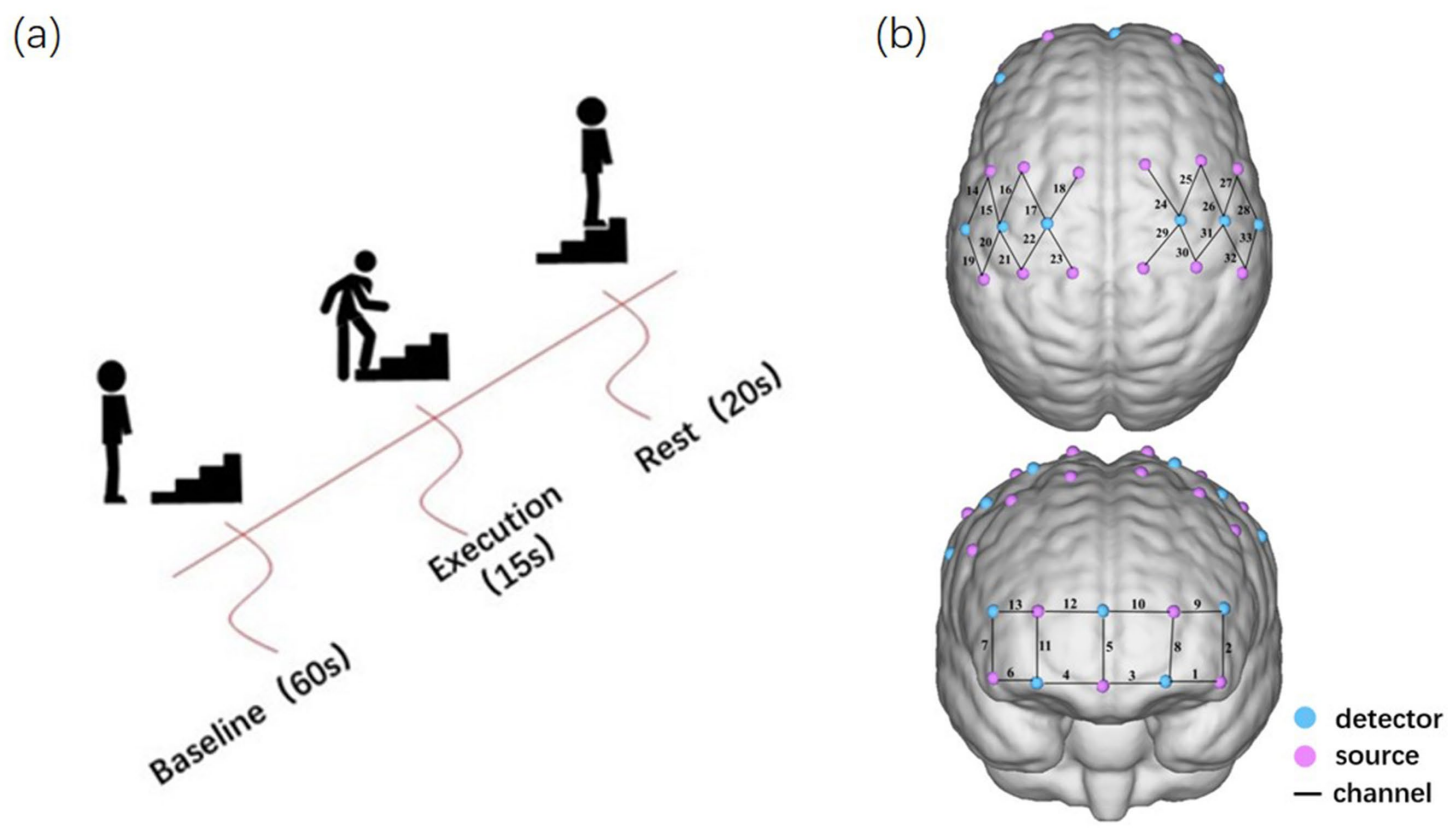
Experimental design. **(A)** Experimental procedure for the repetitive upstairs task. **(B)** The arrangement of optodes and channels.

We performed task prompts with computerized voice prompts to minimize distractions such as movement and sound that may affect brain activity. Before the actual experiment began, participants were briefly introduced to the experimental protocol (Figure 1A). A standardized motor task was that after an initial rest of 1 min to stabilize the baseline, the patient started the repetitive upstairs task. Participants are instructed to ascend the stairs at their normal walking pace, maintaining a uniform rate of ascent. No assistance was allowed during the task, and an experimenter followed the patient closely to prevent them from falling down. They continued to go up the stairs for 15 s. Then, after a rest of 20 s, the upstairs task was repeated for 15 s; a total of 4 sets were done. We measured the change in OxyHb throughout the task duration in real time after standing still for 1 min.

### 2.4 fNIRS measurement and preprocessing

In this experiment, NirSmartII-3000A equipment (Danyang Huichuang Medical Equipment Co., Ltd., China) was utilized to acquire hemodynamic information from the cortices of the participants. In total, 17 light-source probes and 11 detectors were arranged to form 33 measurement channels, and the channel distance of 3 cm spacing was adopted. Referring to the EEG International 10–20 system for positioning, the frontal and parietal cortex were covered (Figure 1B).

Preprocessing was conducted using MATLAB 2016 (MathWorks, Inc., Natick, MA, United States). A spline interpolation method was employed to detect and eliminate motion artifacts with a selected standard deviation threshold of six and a peak threshold of 0.5. Subsequently, physiological noise from heartbeat, respiration, and low-frequency machine noise were filtered using a Butterworth band-pass filter of 4th order 0.01–0.2 Hz. Finally, the path difference factor was set to [6 6], and the relative concentration of oxygenated hemoglobin (HbO) was calculated according to the modified Beer–Lambert law. Only the evoked response of HbO was analyzed in this study because of its high signal-to-noise ratio. A general linear model (GLM) was employed to compute the *β*-values associated with the repetitive upstairs tasks, with the *β*-value serving as a measure of activation in the corresponding channel region.

### 2.5 Statistics

In this study, we utilized SPSS 26.0 statistical software for data analysis. For continuous variables that conformed to a normal distribution, a paired-samples *t*-test was used to compare the *β*-values of the repetitive upstairs task with the null hypothesis that the population mean was zero (resting state) as well as to compare the patients’ pre- and post-injury Lysholm knee scores. In addition, the differences in *β*-values between the patient group and the control group during the task were analysed using an independent-samples *t*-test. This comparison aims to ascertain the presence of significant activation pathways in patients with ACLR during the repetitive upstairs task. Conversely, for continuous data that do not conform to a normal distribution, the Wilcoxon signed-rank test was employed. According to the Bonferroni correction method, when simultaneously testing independent hypotheses on the same dataset, the statistical significance level for each hypothesis should be adjusted to 1/*n* of the significance level used for testing a single hypothesis. Since the primary focus is on comparing the *β*-values among three groups: ACLR group (exercise vs. rest), control group (exercise vs. rest), and ACLR group (exercise vs. control), a *p*-value less than 0.0167 (0.05/3) is considered statistically significant. For categorical data, the chi-squared test was employed, while general statistical analysis was performed with a significance threshold set at *p* < 0.05. Furthermore, Pearson correlation analysis was used to assess the relationship between *β*-values and the Lysholm Knee Scale scores, with p < 0.05 indicating statistical significance.

## 3 Results

**Figure 2:**
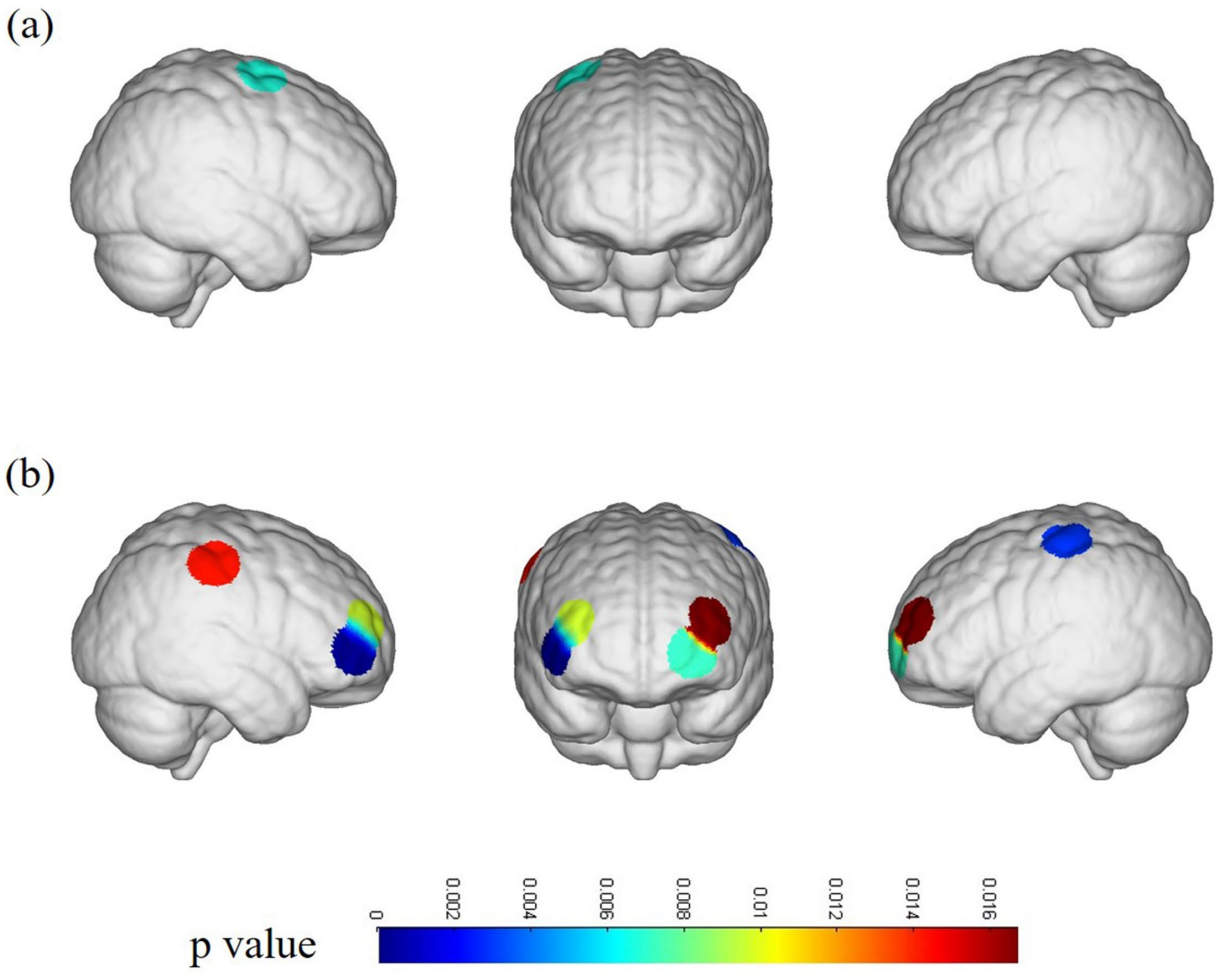
fNIRS channel activation map. **(A)** Channel activation in patients with ACLR under the repetitive upstairs task. **(B)** Channel activation in healthy subjects under the repetitive upstairs task.

**Figure 3:**
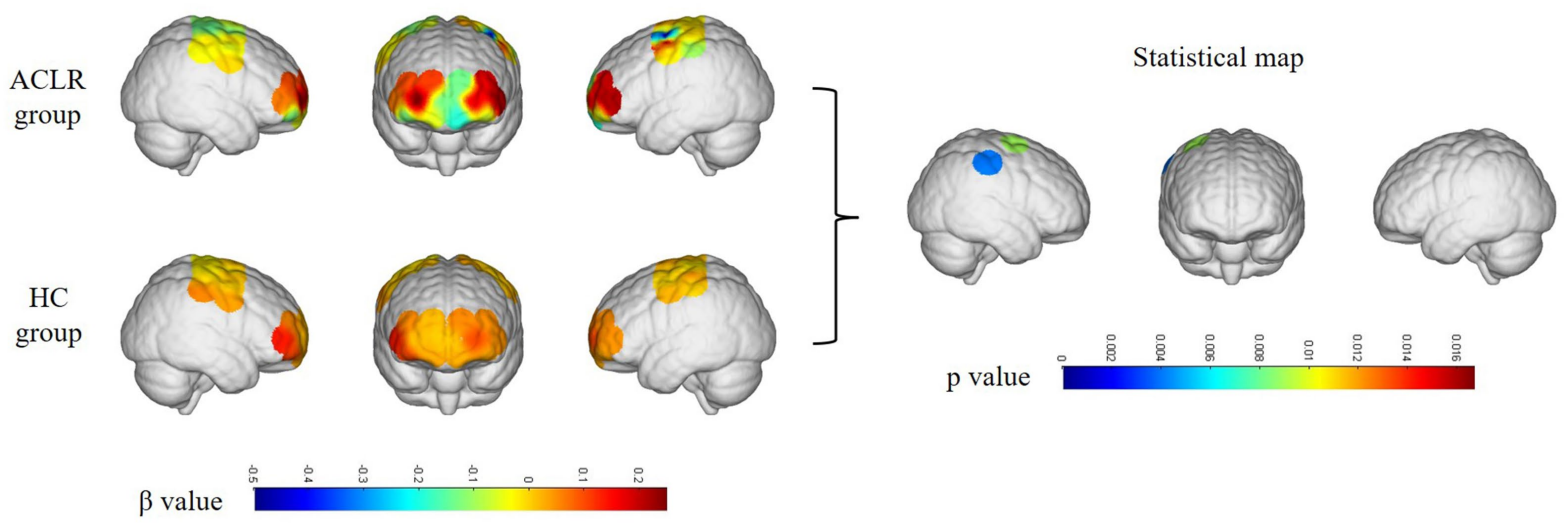
Differences in channel activation between the ACLR group and healthy controls under the repetitive upstairs task. ACLR, anterior cruciate ligament reconstruction; HC, healthy control.

In the ACLR group, *β*-values in channel 25 (corresponding to the pre-motor and supplementary motor cortex) (*t* = 4.0461, *p* = 0.0067) demonstrated a significant negative change during the repetitive upstairs task (Figure 2A) (*t*-values and *p*-values for each channel are shown in Table 2). Conversely, in the healthy control group, significant increases in *β*-values were observed in frontal polar regions corresponding to Channel 6 (*t* = −3.0489, *p* = 0.0066), Channel 7 (*t* = −4.5723, *p* = 0.0002), Channel 8 (*t* = −3.0089, *p* = 0.0072), Channel 13 (*t* = −2.8789, *p* = 0.0096), as well as in the primary somatosensory cortex represented by Channel 20 (*t* = −3.4200, *p* = 0.0029) and Channel 33 (*t* = −2.6974, *p* = 0.0143) (Figure 2B) (*t*-values and *p*-values for each channel are shown in Table 3). When comparing the two groups, the ACLR group exhibited significantly lower *β*-values in Channel 25 (*t* = 2.7583, *p* = 0.0089) during the task, while the healthy control group showed significantly higher *β*-values in Channel 33 (*t* = 3.0618, *p* = 0.0040) (Figure 3) (*t*-values and *p*-values for each channel are shown in Table 4).

**Table 2:** Comparison of *β*-values in different brain regions and corresponding channels in ACLR group repeating the upstairs task.

Cerebral area	Channel	Means ± S.D.	*t*	*p*
Orbitofrontal area	1	−0.0643 ± 0.2878	0.5914	0.5759
	3	−0.1895 ± 0.2628	1.9076	0.1051
	4	−0.0522 ± 0.5025	0.2746	0.7929
	6	−0.1325 ± 0.2995	1.1701	0.2863
Frontopolar area	2	0.2123 ± 0.3472	−1.6183	0.1567
	5	−0.1333 ± 0.2311	1.5255	0.1780
	7	0.0652 ± 0.3848	−0.4481	0.6698
	8	0.1690 ± 0.4597	−0.9728	0.3682
	9	0.1760 ± 0.4488	−1.0376	0.3395
	10	−0.1359 ± 0.4339	0.8285	0.4391
	11	0.2477 ± 0.5380	−1.2184	0.2688
	12	0.1118 ± 0.1545	−1.9146	0.1040
	13	0.1002 ± 0.2727	−0.9720	0.3686
Pre-motor and supplementary motor cortex	14	−0.0206 ± 0.3797	0.1432	0.8908
	15	0.1926 ± 0.4405	−1.1568	0.2913
	16	−0.4998 ± 1.1688	1.1315	0.3010
	17	−0.0197 ± 0.1031	0.5062	0.6308
	18	0.0319 ± 0.1802	−0.4686	0.6559
	24	−0.0586 ± 0.1623	0.9559	0.3760
	25	−0.1469 ± 0.0961	4.0461	0.0068^**^
	26	0.0036 ± 0.1947	−0.0489	0.9626
	27	−0.0580 ± 0.2084	0.7366	0.4892
	28	−0.0019 ± 0.3166	0.0159	0.9878
	29	−0.1116 ± 0.2034	1.4515	0.1968
	30	−0.1420 ± 0.3813	0.9853	0.3625
Primary somatosensory cortex	19	−0.1035 ± 0.1543	1.7745	0.1263
	20	−0.0067 ± 0.1999	0.0890	0.9320
	21	−0.0056 ± 0.2630	0.0564	0.9569
	31	−0.1427 ± 0.2627	1.4377	0.2006
	32	−0.0111 ± 0.2235	0.1317	0.8995
	33	−0.0379 ± 0.2906	0.3453	0.7416
Primary motor cortex	22	−0.0165 ± 0.2452	0.1784	0.8643
	23	−0.0265 ± 0.3424	0.2046	0.8446

ACLR, anterior cruciate ligament reconstruction; means ± S.D., means ± standard deviation. Comparison with the overall mean of 0 samples, ^**^indicates *p* < 0.0167.

**Table 3:** Comparison of *β*-values in different brain regions and corresponding channels in healthy control group repeating the upstairs task.

Cerebral area	Channel	Means ± S.D.	*t*	*p*
Orbitofrontal area	1	0.0309 ± 0.0947	−1.4609	0.1604
	3	0.0278 ± 0.0860	−1.4456	0.1646
	4	0.0326 ± 0.0811	−1.7964	0.0883
	6	0.1020 ± 0.1496	−3.0489	0.0066^**^
Frontopolar area	2	0.0478 ± 0.0899	−2.3785	0.0280
	5	0.0142 ± 0.1029	−0.6166	0.5448
	7	0.1371 ± 0.1341	−4.5723	0.0002^**^
	8	0.0993 ± 0.1476	−3.0089	0.0072^**^
	9	0.0497 ± 0.1010	−2.2006	0.0403
	10	0.0538 ± 0.0961	−2.5054	0.0215
	11	0.0186 ± 0.1023	−0.8124	0.4266
	12	0.0199 ± 0.0790	−1.1253	0.2745
	13	0.0528 ± 0.0820	−2.8789	0.0096^**^
Pre-motor and supplementary motor cortex	14	0.0253 ± 0.0891	−1.2717	0.2188
	15	−0.0124 ± 0.0576	0.9597	0.3492
	16	−0.0020 ± 0.0981	0.0913	0.9282
	17	0.0089 ± 0.0693	−0.5722	0.5739
	18	0.0308 ± 0.0764	−1.8049	0.0870
	24	0.0118 ± 0.0795	−0.6647	0.5142
	25	−0.0174 ± 0.0896	0.8696	0.3954
	26	0.0188 ± 0.0959	−0.8781	0.3908
	27	−0.0044 ± 0.0895	0.2218	0.8268
	28	0.0401 ± 0.1056	−1.6983	0.1058
	29	−0.0368 ± 0.0777	2.1174	0.0476
	30	−0.0154 ± 0.1061	0.6510	0.5228
Primary somatosensory cortex	19	0.0005 ± 0.0683	−0.0299	0.9765
	20	0.0485 ± 0.0634	−3.4200	0.0029^**^
	21	−0.0210 ± 0.0627	1.4965	0.1510
	31	−0.0277 ± 0.0825	1.5021	0.1495
	32	−0.0083 ± 0.0826	0.4489	0.6586
	33	0.0583 ± 0.0966	−2.6974	0.0143^**^
Primary motor cortex	22	−0.0203 ± 0.0631	1.4390	0.1664
	23	0.0143 ± 0.0892	−0.7144	0.4836

Means ± S.D., means ± standard deviation. Comparison with the overall mean of 0 samples, ^**^indicates *p* < 0.0167.

**Table 4:** Comparison of *β*-values in various brain regions and corresponding channels between the ACLR group and the healthy control group repeating the upstairs task.

Cerebral area	Channel	*t*	*p*
Orbitofrontal area	1	−0.6376	0.5276
	3	2.3856	0.0221
	4	1.3972	0.1705
	6	1.4093	0.1669
Frontopolar area	2	0.7272	0.4716
	5	1.3718	0.1782
	7	1.1571	0.2545
	8	−0.1094	0.9134
	9	−0.5171	0.6081
	10	1.2507	0.2187
	11	−1.5965	0.1187
	12	0.6716	0.5059
	13	0.7814	0.4394
Pre-motor and supplementary motor cortex	14	−0.1336	0.8945
	15	−1.7028	0.0968
	16	1.6132	0.1150
	17	0.7252	0.4728
	18	1.3943	0.1713
	24	2.3276	0.0254
	25	2.7583	0.0089^**^
	26	1.9207	0.0623
	27	0.4283	0.6709
	28	−0.2999	0.7659
	29	0.1067	0.9156
	30	0.5174	0.6079
Primary somatosensory cortex	19	−0.5480	0.5869
	20	1.9159	0.0629
	21	0.0578	0.9542
	31	1.4099	0.1667
	32	0.4429	0.6603
	33	3.0618	0.0040^**^
Primary motor cortex	22	0.9027	0.3724
	23	1.4935	0.1436

ACLR, anterior cruciate ligament reconstruction. Comparison with healthy control group, ^**^indicates *p* < 0.0167.

Correlation analysis between the *β*-values in channel 25 on the right side and Lysholm knee scores in the ACLR group revealed no significant association during the repetitive upstairs task. In addition, the Lysholm scores of patients with ACLR showed a significant reduction compared to their pre-injury scores (72.5 ± 6.86 vs. 99.8 ± 0.48, *p* < 0.001).

## 4 Discussion

This study investigated the brain activation characteristics of patients following ACLR during a repetitive upstairs task using fNIRS and compared the results with a healthy control group. Research findings indicate that patients with right ACLR exhibit significant negative activation in the ipsilateral pre-motor cortex, supplementary motor cortex, as well as the primary somatosensory cortex, a characteristic not observed in healthy individuals. Furthermore, we compared the Lysholm scores of patients before and after their injuries and found that there was a marked functional impairment in the knee following the injury. This evidence supports that the alterations in motor capability and brain function observed in these patients are attributable to the ACL injury itself, rather than other underlying factors.

Pre-motor and supplementary motor cortex are located in front of the primary motor cortex and are responsible for controlling certain aspects of movement. These include preparation for movement, sensory orientation during movement, spatial orientation, intrinsic planning of movement, organization of movement sequences before and after executive, and coordination between the two sides of the body. The primary somatosensory cortex is integral to processing sensory feedback from the entire body during movement. It aids the brain in refining and coordinating motor actions, thereby ensuring precise execution of movements and maintaining postural balance and stability. After an ACLR, the CNS may rely more on other sensory sources, such as visual feedback and spatial awareness (14). Changes in the degree of cortical activation may represent an adaptive modification that plays a crucial role in the successful coordination of dynamic tasks (15).

During knee motion in patients undergoing ACLR, the reduced activation in motor execution and planning areas on the injured side may be attributed to extensive unilateral therapy targeting the injured knee joint (16). Given the characteristics of brain control mechanisms, the brain regulates the movement of the contralateral lower limb; however, injury or rehabilitation interventions may lead to functional reorganization within motor areas, aiding movement on the injured side and reducing activation during movement on the healthy side. The observed significant deactivation in motor-related brain regions may also be attributed to altered motor regulation of the knee joint post-ACLR (17).

It is noteworthy that the negative activations observed in the pre-motor and supplementary motor areas in ACLR patients could potentially be associated with underlying callosal inhibitory mechanisms (18). The corpus callosum, which connects the left and right hemispheres, plays a crucial role in the transmission and integration of information. In the context of motor functions, callosal inhibition is essential for the coordinated movement of bilateral limbs. For ACLR patients, the physiological and motor function alterations resulting from ligament injury and subsequent surgery necessitate the involvement of callosal inhibition to ensure the coordinated function of the limbs during activities such as ascending stairs. This involvement, in turn, suppresses the normal activation of ipsilateral motor control regions (19).

In ACLR patients, the specific manifestations of this brain regions mentioned above reflect the phenomenon wherein damage to peripheral tissues induces alterations in the CNS, and conversely, the effects are reciprocal. Recovery of ankle motor function after stroke is influenced by changes in the strength of intra- and interhemispheric functional connectivity (FC) in motor-related regions of the brain, an EEG study shows (20). Research has shown that brain function undergoes alterations following musculoskeletal injuries, such as ACL injuries. Grooms et al. (21) used fMRI to investigate changes in brain function following ACLR. They observed that patients who had undergone ACLR exhibited altered brain activation patterns during knee flexion-extension movements, indicating a shift toward visuomotor strategies. Another study (22) employing EEG examined the neuroplasticity associated with postural control in patients post-ACLR. This study explored theta (4–8 Hz) and alpha-2 (10–12 Hz) oscillatory bands in motor-related brain areas, revealing greater neural inhibition in the ipsilateral pre-motor and supplementary motor cortex of the ACLR group compared to healthy controls during task execution. Our findings align with these results, suggesting that peripheral joint injuries, even after surgical repair, may induce neuroadaptive changes in the central nervous system (11).

In a study utilizing fNIRS (23), researchers compared brain activation patterns in individuals with chronic lateral ankle instability (CLAI) and healthy controls during cognitive-motor dual-task performance. They found significant differences in activation patterns of the pre-frontal cortex and supplementary motor area between CLAI patients and healthy controls when performing the tasks. The observed differences in brain area activation in this study, consistent with our findings, might reflect neural central functional changes due to impaired proprioceptive input following limb injuries, leading to adaptive modulation in the brain. Kluzik et al. (24) discovered that after musculoskeletal injuries, adaptive changes occur within central processing systems, where diminished functional areas are compensated by other regions; this may be one of the reasons for alterations in lower limb kinematics.

In addition, the frontal lobe and bilateral primary somatosensory cortex in the healthy control group also exhibit activation during stair climbing. The prefrontal area plays a critical role in advanced motor planning and execution, while the primary somatosensory cortex is essential for processing proprioceptive and peripheral sensory information. The increased activation of these regions may indicate more efficient motor control strategies and sensory feedback mechanisms in healthy individuals performing repetitive complex motor tasks. Our findings reveal that multiple brain regions are significantly activated during the stair climbing process in healthy individuals, whereas fewer brain regions show significant activation in patients. This suggests that patients exhibit a unique activation pattern during motor tasks and rely more heavily on brain regions associated with motor control and planning to accomplish the stair climbing task.

Following ACLR, residual central and peripheral functional alterations often persist, and traditional rehabilitation approaches may not fully restore normal motor function in all patients. Despite rehabilitation, prolonged deficits in neuromuscular control remain evident (14). Recent studies have highlighted that neuromuscular biophysical enhancement techniques, such as transcranial magnetic stimulation (TMS) and transcranial electrical stimulation (tES), can significantly improve human motor performance. Therefore, applying external interventions like TMS or tES to specific brain regions may be beneficial.

Moreover, the attentional and environmental components of neuromuscular function are largely unaddressed in the current ACL rehabilitation programs. Therefore, during rehabilitation, greater emphasis should be placed on integrating sensory-visual-motor control factors, including reaction time, information processing, attention focus, visual-motor control, and complex task-environment interactions (25).

Finally, based on the distinctive brain area manifestations observed during the repetitive upstairs tasks in ACLR patients, the changes in peripheral functions among these patients are consistently accompanied by corresponding changes of the central nervous system (26). FNIRS can serve as a personalized tool for assessing rehabilitation outcomes following ACLR, enabling the development of individualized rehabilitation exercise programs for patients. Additionally, it provides a theoretical foundation for the application of neurobiomechanical enhancement technologies in rehabilitation. By incorporating neurorehabilitation techniques into the rehabilitation program for ACLR patients, we aim to enhance the rehabilitation process and improve treatment efficacy.

## 5 Summary

In this study, we observed that peripheral changes in limb function induced functional changes in central brain regions, suggesting a potential functional connectivity between these brain regions. In this context, we underscore the importance of neurorehabilitation. In healthy individuals, heightened engagement in various forms of physical activity is associated with greater brain volume, gray matter density, and cortical thickness. Even small increments in additional exercise have been shown to benefit brain health (27). This notion holds particular relevance for the rehabilitation exercises patients undergo following ACLR. fNIRS can serve as a valuable tool for assessing the changes in brain function and the efficacy of rehabilitation following ACL injury.

However, the application of fNIRS to sports injuries is limited. While this study demonstrates the feasibility of fNIRS in exploring the changes in brain function after sports injuries, several challenges remain, including the small sample size, incomplete coverage of brain regions, and the absence of a standardized fNIRS signal processing protocol. Therefore, we anticipate more future fNIRS studies on sports injuries to offer novel approaches to cerebral rehabilitation for patients.

## Data Availability

The raw data supporting the conclusions of this article will be made available by the authors, without undue reservation.
